# Effects of Yoga Interventions on Fatigue: A Meta-Analysis

**DOI:** 10.1155/2012/124703

**Published:** 2012-09-06

**Authors:** Katja Boehm, Thomas Ostermann, Stefania Milazzo, Arndt Büssing

**Affiliations:** Center for Integrative Medicine, Faculty of Medicine, University of Witten/Herdecke, 58239 Herdecke, Germany

## Abstract

*Background*. Researchers aimed at systematically reviewing and meta-analyzing the effectiveness of yoga interventions for fatigue. *Methods*. PubMed/Medline was searched until January 2012 for controlled clinical studies. Two reviewers independently extracted the data. The methodological quality of the studies was assessed. A meta-analysis was performed. *Results*. Nineteen clinical studies (total *n* = 948) were included in this review. Investigated yoga styles included Hatha, Iyengar, Asanas, Patanjali, Sahaja, and Tibetan yoga. Participants were suffering from cancer, multiple sclerosis, dialysis, chronic pancreatitis, fibromyalgia, asthma, or were healthy. Yoga had a small positive effect on fatigue (SMD = 0.27, 59% CI = 0.23–0.31). Seven studies received 4 points on the Jadad score. There were baseline differences in at least 5 studies. *Conclusion*. Overall, the effects of yoga interventions on fatigue were only small, particularly in cancer patients. Although yoga is generally a safe therapeutic intervention and effective to attenuate other health-related symptoms, this meta-analysis was not able to define the powerful effect of yoga on patients suffering from fatigue. Treatment effects of yoga could be improved in well-designed future studies. According to the GRADE recommendations assessing the overall quality of evidence, there is a moderate effect of the confidence placed in the estimates of the effects discussed here.

## 1. Introduction

Prolonged fatigue is defined as self-reported, persistent fatigue lasting 1 month or longer. In the United States it is reported that 24% of the general adult population has had fatigue lasting 2 weeks or longer; 59% to 64% of these persons reported that their fatigue has no defined medical cause [1, 2]. When fatigue cannot be specifically explained by a medical condition such as anemia or hypothyroidism, it may represent a chronic fatigue syndrome. Chronic fatigue syndrome is an illness characterized by profound disabling fatigue lasting at least 6 months (persistent or relapsing) and accompanied by numerous rheumatological, infectious, and/or neuropsychiatric symptoms [3]. The presence of chronic fatigue requires clinical evaluation to identify any conditions underlying or contributing which may require treatment. Further diagnosis cannot be made without such an evaluation. 

Risk factors for fatigue are cancer and its treatment, pain, nausea, depressive symptoms, and other psychological dysfunctions or burdens. All models that try to explain the cause and development of fatigue assume that there are complex, multicausal processes that create the disability. Suggested pathophysiological factors include a dysregulation of inflammatory cytokines, interruption of the regulatory circuits of the hypothalamus, changes in the serotonin system of the central nervous system, and interruption of the circadian secretion of melatonin and of the circadian rhythm [4–15]. 

Among the various treatment strategies, that is, exercise, psychosocial support, stress management, nutrition, sleep regulation, and restorative therapy [34], physical activity was identified as important particularly for patients with advanced stages of cancer to attenuate anxiety, depression, stress, and fatigue [35]. Also, meditation programs were shown to improve mood and sleep quality and to reduce stress and fatigue in cancer patients [36]. Yoga, one of the best known and frequently used mind-body interventions, combines physical exercises and meditation [37] and might thus also be effective to attenuate fatigue.

Yoga's conceptual background originates in Indian philosophy, and there are numerous schools or types of yoga (i.e., Iyengar, Viniyoga, Sivananda, etc.) with distinct priorities in terms of spiritual and physical practices [37]. A typical yoga session with a specific sequence of postures (*asanas* of Hatha Yoga), breathing techniques (*pranayama*), and mental concentration/meditation (*dhyana*) lasts between 1 and 2 hours.

Presumed benefits of yoga include increased muscular strength, flexibility, range of motion, energy, relaxation, and sense of well-being, decreased pain, improved sleep quality, stress reduction, and control over physiological parameters [26, 38–41].

So far, in one RCT it has been demonstrated that the practice of yoga improves fatigue levels in psychiatric inpatients following at least one yoga session [42]. Furthermore, it was shown in a 4-month RCT that yoga could reduce fatigue scores in asthma patients [25] and a 12-week intervention with an RCT design also reduced fatigue scores in patients with chronic pancreatitis [43]. This range of health conditions shows that yoga is being used as a therapy for patients with numerous conditions, some of which involve fatigue.

Thus, yoga could in fact be a beneficial supportive intervention to attenuate fatigue, but there is currently lack of an adequate meta-analysis to assess its effectiveness with respect to fatigue symptoms. To assess its putative relevance in the treatment of patients with fatigue caused by various conditions, we performed a meta-analysis of the current literature focusing on fatigue and fatigue-associated disability. 

## 2. Materials and Methods

### 2.1. Literature Search Strategy

We searched the electronic literature database PubMed/Medline until January 2012 for clinical studies focusing on yoga interventions and fatigue. English language search terms were “yoga * fatigue.” To increase the chance to find all relevant publications describing the effects of yoga interventions on fatigue, there were no limitations in the initial search in terms of language, year, status, or design. Reference lists of reviews on yoga for fatigue were scanned for further trials. Finally, experts were contacted for gray literature not listed in the databases mentioned above, and reference lists of relevant articles and authors were checked. 

### 2.2. Selection Criteria

All potentially eligible studies were retrieved and the full-text articles were reviewed to determine whether they met the inclusion criteria.

Inclusion criteria were controlled clinical studies (randomized or nonrandomized) addressing the effects of yoga interventions on fatigue symptoms. The findings were analyzed with respect to fatigue scores on various outcome measures. We excluded case series, case reports, studies lacking a control group, expert statements, and theoretical reflections. We also excluded studies with complex interventions such as mindful-based stress reduction (MBSR) programs (which include yoga practices), because the contributing effects of the relevant elements cannot be distinguished. 

### 2.3. Data Extraction

Review authors (S. M. and A. B.) assessed studies for inclusion in the review. They took part in the extraction of data and independent assessment of methodological quality (S. M. and K. B.). Disagreements were resolved by consensus. We extracted study data on the following topics: general study design (prospective, multicenter, etc.), treatment allocation (randomization, matched pairs, etc.), treatment concealment and blinding, treatments (yoga style and practices, duration and frequency, type of control intervention), patient characteristics (mean age, gender distribution), diagnosis, adherence to therapy (compliance, drop-outs, etc.), and outcome assessments, that is, fatigue scores pre-post. 

To assess the methodological quality of the respective studies, we adopted the Jadad score which refers to randomization (0 to 2 points), blinding of the assessor (statistician, physician, assessor, or researcher, as cited in the original publications; 0 to 1 points), and dropout reporting (0 or 1 point) as indicators of methodological quality of a study [44]. Because it is impossible to blind also patients (double blind) in yoga studies, the maximum achievable Jadad score was 4 in our review. However, while it is clear what blinding of a “statistician” means, it is not very clear what blinding of “researcher” (as it was stated in some studies) may mean; we assumed that this term refers to the outcome assessor. 

 Allocation concealment was assessed in accordance with the Cochrane guidelines [45]: *A* means adequate (telephone randomization or using consecutively numbered, sealed, opaque envelopes); *B* means uncertainty about the concealment (method of concealment is not known); *C* means inadequate (e.g., alternate days, odd/even date of birth, hospital number).

### 2.4. Statistical Analysis

All relevant outcome data were extracted as they were given in the publication. They were converted into standardized mean differences (SMD) and their standard errors (SE) using standard formulas [45]. An SMD 0.2 indicates a small effect, 0.5 a moderate, and 0.8 a large effect [46].

Overall estimates of the treatment effect were obtained from random effects meta-analysis [47]. We performed various subgroup analyses with respect to condition, methodological quality, and duration of treatment. Heterogeneity between studies was assessed by standard *χ*²-tests and the *I*² coefficient which measures the percentage of total variation across studies due to true heterogeneity rather than chance [48]. *I*² coefficients 25% would indicate low, 30–60% moderate, and >75% high heterogeneity. 

Heterogeneity was formed by study quality (high: Jadad score = 4 and allocation concealment = *A*), moderate quality (scores 2-3), low quality (score 0-1), and type of control group (waiting list: routine care only; active treatment: any other intervention given additionally to routine care). Funnel plot asymmetry was assessed by Egger's test [31].

Finally, we carried out a grading of recommendations assessment, development, and evaluation (GRADE) in order to being able make judgments about evidence and recommendations in this field of yoga for fatigue. The GRADE system judges and rates the quality of the evidence underlying the recommendation made in a meta-analysis [50]. During this process factors that affect the strength of a recommendation include the quality of evidence, the uncertainty about the balance between beneficial and harmful consequences, the uncertainty or variability in values and preferences, and the uncertainty about whether the intervention represents a wise use of resources [51]. The quality assessment includes factors such as the design of the study, rating the level of quality and its limitations, rating the consistency across included studies, and the directedness of outcome measures [52].

## 3. Results 

### 3.1. Literature Search Results

**Figure 1:**
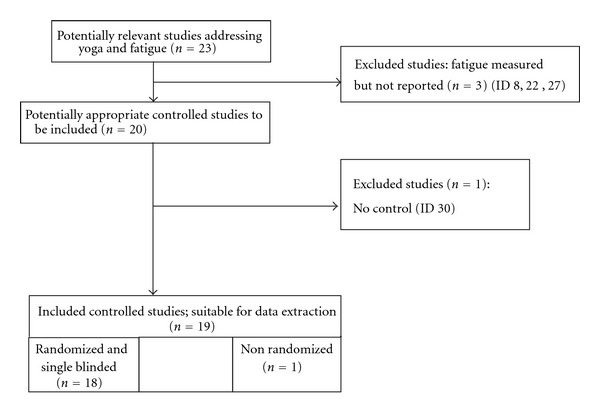
Flow chart.

We found 23 potentially relevant studies addressing the effects of yoga on fatigue [16–23, 27–30, 32, 33, 49, 53–59]. Among them, four studies were excluded because either they delivered no baseline data, had no control group, or measured but did not report pre-post fatigue scores. Nineteen controlled studies with a total of 948 patients were considered eligible for inclusion (Figure 1). 

### 3.2. Participant Characteristics

The type of patients included breast cancer patients (*n* = 8 studies), different types of cancer (*n* = 1), lymphoma (*n* = 1), patients with multiple sclerosis (*n* = 2), dialysis (*n* = 1), chronic pancreatitis (*n* = 1), fibromyalgia (*n* = 1), asthma (*n* = 1), and also healthy participants (*n* = 3). The number of individuals analyzed varied considerably from 14 to 135 (mean ± SD: 62 ± 35) (Table 1). 

### 3.3. Study Designs and Methodological Quality

According to specifications in the articles, all 19 studies had a prospective design. Eighteen studies were randomized, whereas one study was not randomized. Seven studies were single blinded (i.e., statistician, physician, assessor, or researcher, as stated in the respective studies) and randomized and thus had a higher methodological quality (JADAD score 4). Ten studies were randomized (without blinding) and displayed a moderate methodological quality (scores 2-3). One study was randomized but with a low quality (Jadad score 1) (Table 1). 

Eleven studies applied a waiting-list control group design of which 2 others also had a third active control group either attending an exercise class using a stationary bike or a walking exercise class. Two further studies applied a design where one control group received standard care and one of these 2 trials also had an active control group which received supportive counseling. Other active controls were a health education and a physical education class, sports climbing, swimming, relaxation, and cognitive behavioral therapy.

The methodological quality of the studies ranged from very low to medium. Two clinical trials only received one point on the Jadad score, five studies received 2 points, 5 studies received 3 points, and 7 further studies received the maximum of 4 points. Ten studies were assuring blinding of the outcome assessor, which means that in the interpretation of the remaining studies a detection bias should be taken into account. Only 7 studies addressed incomplete outcome data, meaning that rest of the trials is under the influence of attrition bias. Twelve studies analyzed their data based on intention to treat. Apart from 2 trials all others described the dropout rate and how the data were dealt with. 

### 3.4. Intervention Characteristics: Duration of Intervention

Researchers in those included studies applied Hatha, Iyengar, Asanas, Patanjali, Sahaja, and Tibetan yoga. The whole duration of taught yoga classes lasted between 5 and 24 weeks with the majority of studies lasting more than 8 weeks. 

### 3.5. Outcome Measures

Most of these studies measured more symptom outcomes than merely fatigue, for instance, anxiety, depression, and sleep disturbance. Instruments to measure fatigue symptoms were MFI, POMS, SF-36, FACT-F, EORTC, BFI, diaries, and visual analogue scales (VAS).

### 3.6. Effect Sizes Fatigue

**Figure 2:**
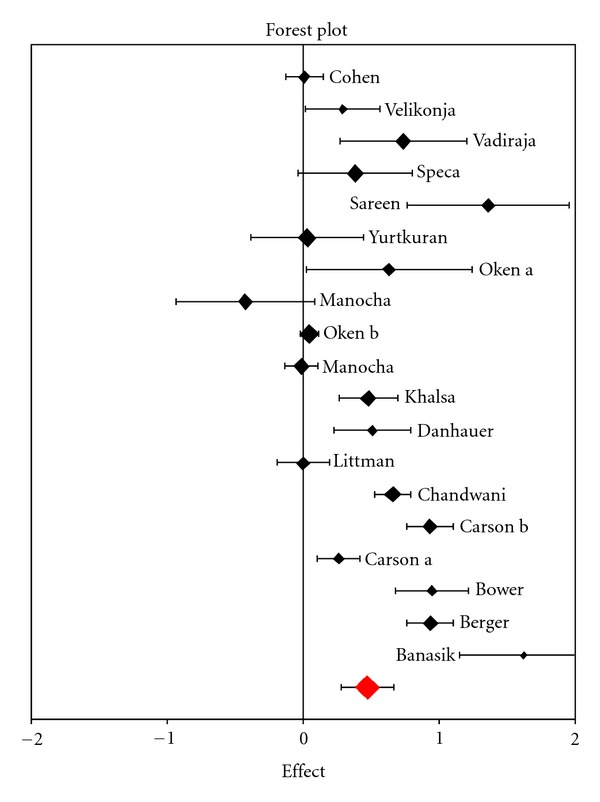
Forest plot with effect sizes for included studies, SMD = 0.27 [0.23–0.31], *I* ^2^ = 94.4%.

As shown in Table 1, most studies reported positive effects in favor of the yoga interventions. With respect to fatigue, a random effect meta-analysis estimated the overall treatment effect at SMD = 0.27 [0.23; 0.31] ranging from −0.43 ± 0.26 to 1.62 ± 0.24 (Figure 2). Overall heterogeneity of study results was high (*I* ^2^ = 94.4%).

### 3.7. Subgroup Analyses

Subgroup analysis (Table 2) enrolling only those studies with comparable means at baseline showed effect sizes ranging from −0.4 ± 0.23 to 1.6 ± 0.24. Those studies with a high methodological quality showed less of an effect of the yoga intervention on fatigue (SMD = 0.24 [0.18; 0.29]) than those with high quality (SMD = 0.46 [0.37; 0.55]).

Trials with a waiting list control group also showed less of an effect (SMD = 0.22 [0.18; 0.27]) than those that used other controls such as, for instance, health education class, relaxation, swimming, or exercise (SMD = 0.47 [0.38–0.55]) (Table 2).

Interestingly, studies enrolling (breast) cancer patients had lower effects in favor of yoga than those studies with other conditions (including healthy individuals) (Tables 1 and 2). 

### 3.8. Nonrandomized Study

One study was included that was non-randomised. Velikonja et al. [33] carried out a clinical controlled trial with 50 healthy participants who chose either to take part in a Hatha yoga class or to attend a swimming class for a 14-day treatment period. Changes in fatigue were assessed with the POMS and there was a positive effect of the yoga over the swimming (SMD = 0.93 (SD = 0.09)).

## 4. Discussion

The findings of this meta-analysis, enrolling 18 RCT, with single-blinding and 1 N-RCT, indicate small effects of yoga interventions on fatigue, particularly in patients with cancer.

The overall treatment effects were weak, while the heterogeneity was high. Subgroup analyses did not provide further explanations (i.e., studies with passive control had smaller effects that those with an active control; studies with higher quality had better effects than those with low quality). In fact, among 7 studies with a high quality (Jadad score 4), 4 studies had high treatment effects, 1 study a weak effect, and 2 studies no effect. The highest effects were observed in studies with low quality (Jadad score 1-2), but the lowest, too. This may be attributed to the fact that generally adequate sample sizes in the high-quality studies permitted a greater statistical power to detect any beneficial effects of the treatments. Possibly, further sub-group analyses could provide an indication of confounding variables which were not taken into consideration in this meta-analysis.

It was striking that studies enrolling patients with cancer had lower effects than those with other conditions. One may conclude that the effectiveness of yoga interventions to attenuate cancer-related fatigue is limited. Nevertheless, at least four of these studies had strong effects in favor of the yoga interventions, while two studies (and one study enrolling lymphoma patients) showed no beneficial effects compared to the control group. Most of these studies had a passive control, and thus one may expect effects in favor of the yoga group. Moreover, among these studies one may find those with both higher and lower quality, different types of yoga styles, and differences in the duration of treatments (7 to 24 weeks)—and thus one cannot easily attribute the effects to differences in quality of the studies. Because of these less clear-cut data with respect to cancer-related fatigue, more convincing high-quality studies with active controls and circumscribed duration of treatment (i.e., 12 weeks) are highly encouraged to draw valid conclusions. 

With respect to other indications, yoga had weak effects on fatigue in patients with fibromyalgia (SMD 0.26), while in two studies with multiple sclerosis the effects ranged from SMD 0.29 to 0.63. The effects were much better in a study with weak methodology enrolling patients with chronic pancreatitis (SMD 1.36). There were no relevant effects in patients with dialysis and lymphoma. However, three studies addressed fatigue symptoms in healthy individuals; two studies with weak methodology reported positive effects (SMD ranging from 0.48 to 0.93), while one study with higher quality showed no effect in favor of yoga (SMD 0.03). Thus, the evidence that yoga might be effective to treat fatigue symptoms in noncancer diseases is so far rather weak. Although the summarized treatment effects in the cancer studies were weak, there are at least some promising studies which indicate that the treatment effects could be improved. It was of interest that the best results in cancer patients were yielded in those studies with shorter duration of treatment which could indicate that motivation of cancer patients might be a crucial factor too. 

Certainly, this meta-analysis has its limitations. For example, the pooled estimates were based on heterogeneous data, with respect to indications, control groups, duration of treatment, yoga style, and methodological quality of these studies. The time intervals of measuring pre/post-fatigue were different across all studies. Furthermore, one non-randomized study was included in the meta-analysis (Velikonja et al., [33])—albeit discussed separately. A general problem in this field of research also seems to be the variety of outcome measures used for the given endpoint—in this case fatigue symptoms. For instance, the “vitality” subscale on the SF-36 does not adequately capture fatigue since the used items do not capture the full burden of chronic fatigue, which is reflected by the developing research team who defines minimum vitality as “feeling tired and not as fatigued.” With respect to the GRADE criteria (Table 3), there were neither serious risks of bias nor serious inconsistency, indirectness, nor imprecision. Overall, the estimated treatment effect of the papers was moderate, but the studies' outcome effects were not associated with their methodological quality. 

Future studies should also address patients' motivation, impacts not only the number of drop-outs, but also the (intrinsic) intensity of practice (“inner congruence”). These studies have to identify which patients may benefit from the interventions [60] and which aspects of the yoga interventions (i.e., physical activity and/or meditation and subsequent life style modification) or which specific yoga styles were more effective than others.

## 5. Conclusion

Although yoga is in general a safe therapeutic intervention and effective to attenuate several other health-related symptoms, this meta-analysis is not able to define relevant effects of yoga on patients suffering from fatigue. According to the GRADE recommendations assessing the overall quality of evidence, there is a moderate confidence effect of the confidence placed in the estimates of the effects discussed here (Table 3). Nevertheless, there are some studies on cancer-related fatigue which indicate that treatment effects of yoga could be improved in well-designed future studies.

## Figures and Tables

**Table 1:** Overview of identified studies.

First Author [Study-ID]	Year	Fatigue condition	*N* (analyzed)	Yoga style	Control intervention(s)	Duration of treatment	Methodological quality*	Instrument	SMD	SD
Bower et al. [4]	2011	Breast cancer	31	Iyengar	Health education class	12 weeks	4	FSI	0.95	0.14
Carson et al. [16]	2009	Breast cancer	30	Yoga awareness	Waiting list	8 weeks	4	Diary	0.93	0.09
Chandwani et al. [17]	2010	Breast cancer	61	Patanjali	Waiting list	6 weeks	3	BFI	0.66	0.07
Littman et al. [18]	2011	Breast cancer	63	Hatha	Waiting list	24 weeks	3	FACT-F	−0.01	0.06
Vadiraja et al. [19]	2009	Breast cancer	75	Asanas, Pranayama	Supportive counseling	6 weeks	3	EORTC	0.74	0.24
Banasik et al. [20]	2011	Breast cancer	14	Iyengar	Waiting list	8 weeks	2	Cella's FACT-B	1.62	0.24
Danhauer et al. [21]	2009	Breast cancer	27	Asanas, Pranayama	Waiting list	10 weeks	2	FACT-F	0.51	0.14
Moadel et al. [22]	2007	Breast cancer	128	Hatha	Waiting list	12 weeks	2	FACT-F	0.05	0.03
Carson et al. [23]	2010	Fibromyalgia	50	Yoga awareness	Waiting list	8 weeks	4	FIQ-R	0.26	0.08
Cohen [24]	2004	Lymphoma	39	Tibetan	Waiting list	7 weeks	4	BFI	0.00	0.10
Manocha et al. [25]	2002	Asthma	59	Sahaja	Relaxation, group discussion, CBT	16 weeks	4	POMS		
Oken et al. [26]	2004	Multiple sclerosis	57	Iyengar	Waiting list/Exercise class + stationary bike	24 weeks	4	MFI	0.63	0.31
Oken et al. [27]	2006	Healthy	135	Hatha	Waiting list/Exercise class + walking	24 weeks	4	MFI	0.03	0.21
Speca et al. [28]	2000	Different types of cancer	90	Yoga stretches	Waiting list	7 weeks	3	POMS	0.38	0.21
Yurtkuran et al. [29]	2007	Dialysis	40	Asanas	Standard therapy	12 weeks	3	VAS	0.01	0.07
Khalsa et al. [30]	2011	Healthy	121	Yoga education class	Physical education class	11 weeks	2	POMS	0.48	0.11
Sareen et al. [31]	2007	Chronic pancreatitis	52	Iyengar	Standard therapy	12 weeks	2	SF-36	1.36	0.30
Berger et al. [32]	1992	Healthy	50	Hatha	Swimming	14 weeks	1	POMS	0.93	0.09
Velikonja et al. [33]	2010	Multiple sclerosis	nr	Hatha	Sports climbing	10 weeks	1	MFI	0.29	0.14

*Jadad score.

**Table 2:** Subgroup analyses.

Subgroup	Enrolled studies (ID numbers)	SMD	95% CI	*I* ^2^
Overall	1, 2, 4, 5, 7, 11, 12, 14, 16, 17, 19, 21, 24, 25, 28, 29, 31, 32, 33	0.27	0.23–0.31	94.4%
Jadad score				
High	4, 5, 16, 17, 25, 29, 32	0.46	0.37; 0.55	93.3%
Intermediate	7, 12, 19, 24, 28	0.23	0.15; 0.30	94.3%
Low	1, 2, 11, 14, 21, 31, 33	0.24	0.18; 0.29	96.0%
Control group*				
Waiting list	1, 4, 5, 7, 12, 14, 25, 28, 31	0.22	0.18; 0.27	95.8%
Other controls	2, 11, 19, 21, 24, 29, 32, 33	0.47	0.38; 0.55	93.7%
Condition				
Cancer	1, 4, 7, 12, 14, 19, 25, 28, 29, 31	0.20	0.15; 0.24	94.3%
Noncancer	2, 5, 11, 16, 17, 21, 24, 32, 33	0.46	0.24; 0.67	76.6%

*Excluded from analysis since more than 1 control (Oken et al. [27], Oken et al. [49]).

**Table 3:** GRADE recommendation: Fatigue (assessed with: various fatigue subscales).

Quality assessment							No of patients (yoga: control)	Effect SMD (95% CI)	Quality
No. of studies	Design	Risk of bias	Inconsistency	Indirectness	Imprecision	Other considerations
19	RCT	No serious risk of bias	Serious^1^	No serious indirectness	No serious imprecision	None	458 : 490	SMD = 0.27, 95% CI = 0.23–0.31, *I* ^2^ = 94.4%	Moderate

^1^Baseline differences between groups in at least 5 studies.
